# Iranian Multi-center Osteoporosis Study (IMOS), 2021–2022: the study protocol

**DOI:** 10.1186/s12877-022-03532-3

**Published:** 2022-10-23

**Authors:** Kazem Khalagi, Noushin Fahimfar, Fatemeh Hajivalizadeh, Mahnaz Sanjari, Mohammad Javad Mansourzadeh, Safoora Gharibzadeh, Gita Shafiee, Koorosh Kamali, Farshid Alaeddini, Farshad Farzadfar, Samaneh Mohseni, Nazli Namazi, Farideh Razi, Kobra Gorgani, Katayoun Kateb Saber, Nekoo Panahi, Ramin Heshmat, Alireza Raeisi, Bagher Larijani, Afshin Ostovar

**Affiliations:** 1 Osteoporosis Research Center, Endocrinology and Metabolism Clinical Sciences Institute, Tehran University of Medical Sciences, No 10, Jalale Al Ahmad St., Next to Dr. Shariati Hospital Complex, Tehran, 1411713137 Iran; 2 Obesity and Eating Habits Research Center, Endocrinology and Metabolism Clinical Sciences Institute, Tehran University of Medical Sciences, Tehran, Iran; 3 Department of Epidemiology and Biostatistics, School of Public Health, Tehran University of Medical Sciences, Tehran, Iran; 4 Center for Non-Communicable Disease Control & Prevention, Deputy of Public Health, Ministry of Health and Medical Education, Tehran, Iran; 5 Prevention of Metabolic Disorders Research Center, Research Institute for Endocrine Sciences, Shahid Beheshti University of Medical Sciences, Tehran, Iran; 6 Department of Epidemiology and Biostatistics, Pasteur Institute of Iran, Tehran, Iran; 7 Chronic Diseases Research Center, Endocrinology and Metabolism Population Sciences Institute, Tehran University of Medical Sciences, Tehran, Iran; 8 Social Determinants of Health Research Center, Zanjan University of Medical Sciences, Zanjan, Iran; 9 Tehran Heart Center, Tehran University of Medical Sciences, Tehran, Iran; 10 Non-Communicable Diseases Research Center, Endocrinology and Metabolism Population Sciences Institute, Tehran University of Medical Sciences, Tehran, Iran; 11 Takapo Teb Corp, Tehran, Iran; 12 Diabetes Research Center, Endocrinology and Metabolism Clinical Sciences Institute, Tehran University of Medical Sciences, Tehran, Iran; 13 Metabolomics and Genomics Research Center, Endocrinology and Metabolism Molecular-Cellular Sciences Institute, Tehran University of Medical Sciences, Tehran, Iran; 14 Metabolic Disorders Research Center, Endocrinology and Metabolism Cellular and Molecular Institute, Tehran University of Medical Sciences, Tehran, Iran; 15 School of Medicine, Shiraz University of Medical Sciences, Shiraz, Iran; 16 Endocrinology and Metabolism Research Center, Endocrinology and Metabolism Clinical Sciences Institute, Tehran University of Medical Sciences, Tehran, Iran

**Keywords:** Osteoporosis, Sarcopenia, Prevalence, National Survey, Iran, Study protocol

## Abstract

**Background:**

This paper presents the protocol of the 4^th^ round of Iranian Multi-center Osteoporosis Study (IMOS), a national survey with the primary objective of estimating the prevalence of osteoporosis and sarcopenia and their risk factors in a representative sample of urban and rural populations.

**Methods:**

The target population of the survey is all individuals ≥ 50 years in Iran. A multi-stage random sampling method has been used in the study. We stratified the 31 provinces of the country into 5 strata based on the distribution of their potential risk factors for osteoporosis and randomly selected one or two provinces from each stratum. Then, we invited 2530 people aged ≥ 50 years recruited in the 8^th^ National Survey of None Communicable Diseases (NCD) Risk Factors (STEPs-2021) in the selected provinces to participate in IMOS. Body composition measurements including bone mineral density, muscle mass, and fat mass are measured through Dual-energy X-ray Absorptiometry (DXA) method using HOLOGIC (Discovery and Horizon) devices; and Trabecular Bone Score (TBS) is measured on the DXA scans using iNsight software. Anthropometric measurement and physical examinations are made by a trained nurses and other required information are collected through face-to-face interviews made by trained nurses. Laboratory measurements are made in a central lab. The prevalence of osteoporosis and sarcopenia will be estimated after applying sampling design, non-response, and post-stratification weights to the data.

**Discussion:**

IMOS will provide valuable information on the prevalence and determinants of osteoporosis and sarcopenia at the national level, and the results can be used in evaluating health system interventions and policymaking in the field of musculoskeletal diseases.

## Background

Osteoporosis is an age- and gender-related disease that is characterized with decreased bone density and quality and higher risk of fragility fractures. This silent and asymptomatic disease, along with cancer, myocardial infarction, and stroke, have been introduced as the four main enemies of mankind [1]. Globally, it is estimated that more than 200 million people have osteoporosis [2], and one in three women and one in five men over the age of 50 experience an osteoporotic fracture [3].

The prevalence of osteoporosis among the Iranian elderly aged ≥ 60 years is estimated to be more than 40% [4]. A meta-analysis showed that age-standardized incidence rate of hip fracture in Iranian men and women aged ≥ 50 are 138/100000 and 158/100000, respectively [5]. The health and economic burden of osteoporosis and fragility fracture in Iran are relatively high [6–8]. Furthermore, Iran is among the countries with highly growing elderly populations [9]. Therefore, it is predicted that elderly syndromes like musculoskeletal diseases, in general, and osteoporosis and sarcopenia, in particular, would be major public health problems in the near future imposing huge burden on the people's health and health economy [1].

Iranian Multi-center Osteoporosis Study (IMOS) is a population-based, cross-sectional survey which has been conducted in Iran to estimate the prevalence of osteoporosis and its risk factors in the country [10]. IMOS has been performed three times in different cities of Iran in 2001, 2005, and 2011 [11–15]. Although, the previous rounds of IMOS revealed important aspects of osteoporosis in the country, they were conducted only in urban areas of large cities and had some limitations in design and conduction so that their findings could not be generalizable to the total population of Iran [10].

This article presents the study protocol of the 4^th^ round of IMOS aiming at estimating the prevalence of osteoporosis and a wide range of its determinants in a representative sample of both urban and rural population of Iran. In addition, the prevalence of sarcopenia, another common elderly health problem, will be investigated for the first time in Iran.

## Methods

### Study population and inclusion and exclusion criteria

The target population of the IMOS are all Iranians aged 50 years and over living in the country. The study inclusion criteria are having age 50 years and older, sufficient physical and mental health to participate in the study, and both genders. Subjects with weight more than 150 kg, cystic fibrosis, pregnant women, subjects with prohibition to measure Bone Mineral Density (BMD) (such as bilateral hip replacement), and those went through X-ray imaging with a contrast in the last 2 weeks (if yes, it should be elapsed at least two weeks since the imaging) are excluded from the study.

### Sample size

We calculated the sample size for estimating the prevalence of osteoporosis and sarcopenia. Since the calculated sample size was higher for estimating prevalence of sarcopenia, so we considered it in the study. Bearing in mind the 15% prevalence of sarcopenia in Iran [16], the estimation precision of 3% and the confidence level of 95%, the sample size was obtained using the Cochran's formula and is 550 in each gender. According to the sampling method (stratified clustered systematic random sampling) and taking into account the design effect of 2 and the attrition rate of 15%, the total sample size is 2530 (1265 in each gender).

### Sampling method

**Fig. 1:**
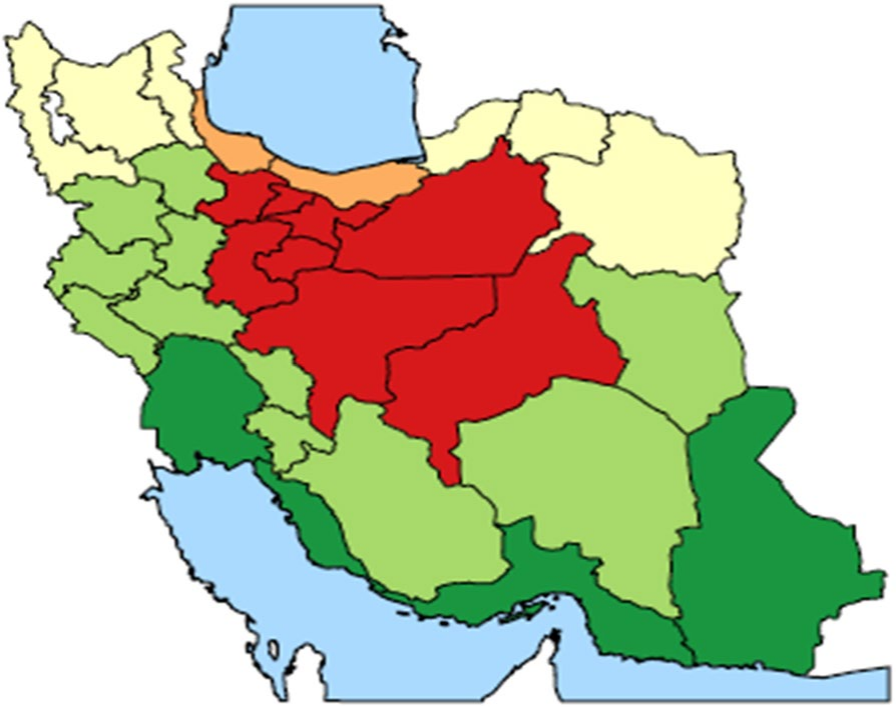
Stratifying the provinces of the country into 5 stratums based on the status of their potential risk factors for osteoporosis (The map depicted by the authors.)

The following multi-stage random sampling method was used in the survey: First, using potential risk factors of osteoporosis such as nutritional status, urbanization index, wealth index, physical activity status, and daily energy consumption, the 31 provinces of the country were stratified into five homogeneous strata using Finite Gaussian Mixture modeling in “mclust” package of R software [17] (see Fig. 1). Then, one or two provinces from each stratum were randomly selected for the study proportional to the size of the ≥ 50 years population of that stratum. In random selection, the most populous provinces in each stratum were given a more chance of recruitment. Accordingly, the provinces of Tehran, Isfahan, Mazandaran, West Azerbaijan, North Khorasan, Fars, Kermanshah, and Khuzestan were selected for the study.

In each of the selected provinces, the study sample included people aged 50 and over participating in the "8^th^ National Survey of None Communicable Diseases (NCD) Risk Factors” (STEPs 2021), which were selected based on the sampling protocol of the STEPs and their questionnaire measurements and physical and laboratory examination were performed in the STEPs [18].

In the STEPs survey, the primary sampling unit was clusters containing 10 eligible individuals. In this study, first, the number of clusters in each province was assigned to the urban and rural areas proportional to their population size. In each area, using the list of households’ zip codes obtained from the post office, the reference household for each cluster was selected by systematic random sampling method. All eligible subjects of these households were included the study. The rest of the required people up to 10 in each cluster were selected from other neighboring households of the reference household by the following method: After the questioner left the house of the reference household, he/she stood behind the main door of the house and the first house on his/her right hand was entered into the study [18].

### Measurements

The measurements of IMOS included two parts: (i) variables measured in the STEPs study; (ii) variables specifically measured in the IMOS.

#### Variables measured in the STEPs study

These measurements were performed by Word Health Organization (WHO) questionnaires (version 3.2) that were translated and standardized in Iran (by assessing the face and content validity and internal consistency) and physical and laboratory examinations. In the STEPs survey, age, sex, education level, socioeconomic status, occupation, marital status, and type of insurance were assessed using demographic questionnaires, and physical activity (WHO Global Physical Activity Questionnaire-2 (GPAQ-2) questionnaire), smoking, alcohol consumption and history of hypertension, diabetes, high total cholesterol, cardiovascular disease, cancer, asthma, and chronic obstructive pulmonary disease were assessed using their respective questionnaires. In this study, physical examinations including blood pressure (2 times), height, weight, waist circumference, hip circumference, heart rate measurements, and pedicometry in 24 h were performed according to the WHO protocol. Also, biochemical tests including total cholesterol, High-Density Lipoprotein (HDL) cholesterol, creatinine, alanine transaminase (ALT), triglycerides, Hemoglobin A1C (HbA1C), and Fasting Plasma Glucose (FPG) were performed through fasting blood sampling and autoanalyzer and laboratory kits approved by the Iranian health reference laboratory [18].

#### The specific IMOS measurements

**Fig. 2:**
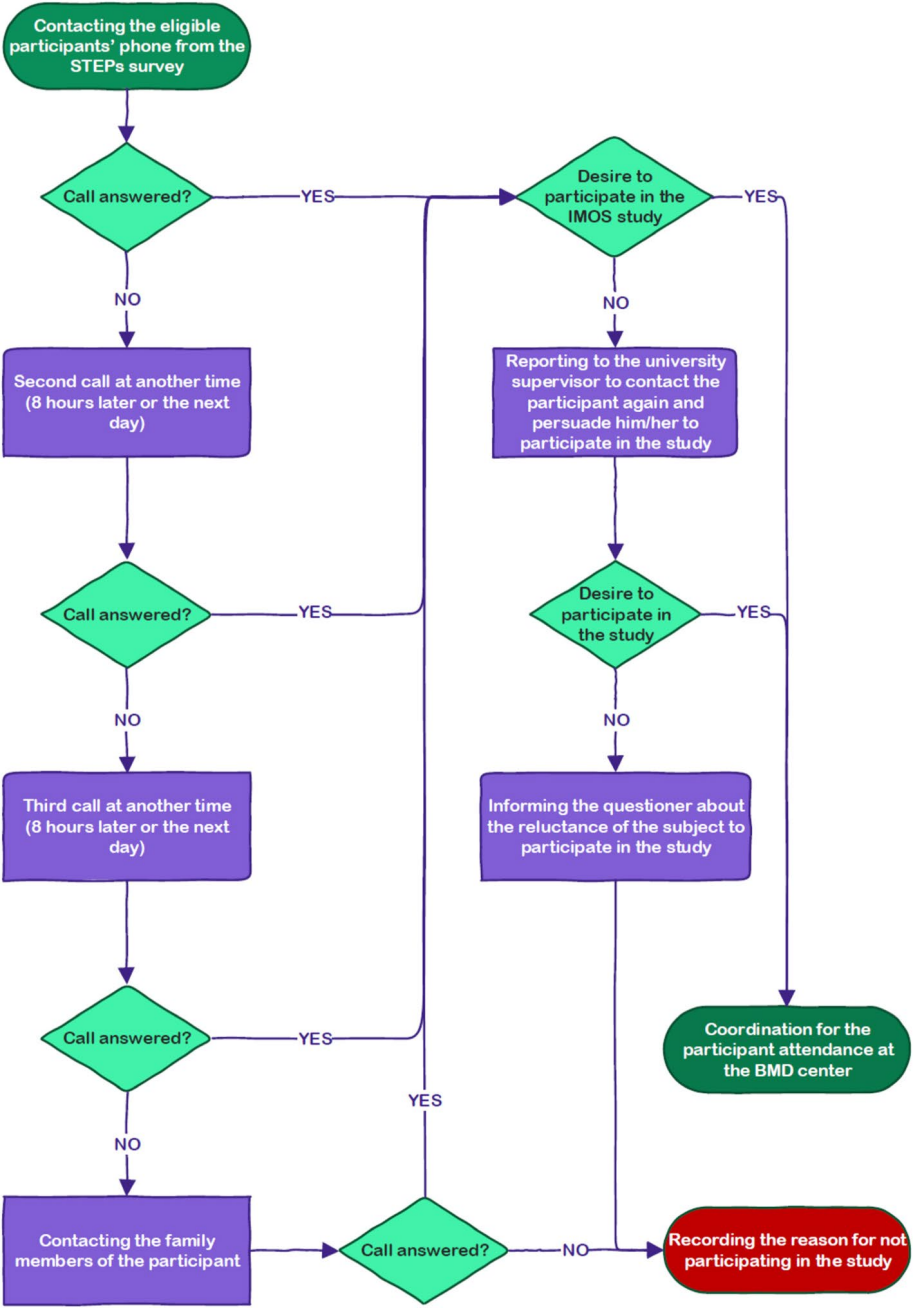
Flowchart of inviting the participants aged 50 and over of steps study to participate in the imos study

Specific measurements of the IMOS study were performed by three methods: questionnaire, physical examination, and BMD. In addition, specific IMOS laboratory tests including serum calcium, phosphorus, vitamin D, Thyroid-Stimulating Hormone (TSH), Parathyroid Hormone (PTH), and albumin were performed on the serum samples stored in the biobank from the same blood samples taken in the STEPs study. The IMOS measurements were taken at BMD centers in selected provinces after inviting people 50 years of age and older who had passed all of the STEPs measurements. After preparing the list of the STEPs participants aged 50 and over, who had completed the three measurement steps in that study, the IMOS questioners invited them according to the flowchart in Fig. 2.

##### Questionnaire

The package of the IMOS specific questionnaires included "Osteoporosis diagnosis", "Osteoporosis treatment adherence", "Fracture and osteoporosis history”, “Fall risk assessment", "Low back pain (Modified Oswestry questionnaire)", "Reproductive history (for women only)", "Muscle health", "Exposure to sunlight", "Knowledge about osteoporosis", "Medical history and use of drugs", "Quality of life ( the12-Item Short Form Health Survey (SF-12) questionnaire)", "Health utilization", and "Food frequency questionnaire" that were asked of the study participants and recorded in the study software on the tablet.

To develop the questionnaires package, first different domains of the study were identified in an expert panel, then appropriate questionnaires for each domain were identified through literature review, and finally the best questionnaire for each domain was selected based on various criteria and expert opinions.

After preparing the initial version of the questionnaire, in several sessions with the expert panel, the text of the questions, possible options in the answer, the logical order of the questions and skipping the questions were assessed. After this stage, the face and content validity and internal consistency and intra-rater reliability of the questionnaires were examined. The content validity of the questionnaire was evaluated qualitatively and quantitatively by 9 experts and the Content Validity Index (CVI) of relevancy, clarity and simplicity and Content Validity Ratio (CVR) of necessity for the questions were calculated (see Table 1) and based on the results, the necessary corrections and changes were made. If the CVI for each question was ≤ 0.80 and the CVR was ≤ 0.78 [19], the content validity of that question was confirmed [20–23]. To evaluate the internal consistency and intra-rater reliability of the questionnaire, the questionnaire was asked of 50 eligible people two times with 2 weeks interval by a trained questioner. After calculating Cronbach's alpha for each questionnaire and Kappa and Intra Class Correlation (ICC) indices (see Table 1), necessary corrections and changes were applied in the questionnaires.

**Table 1:** Results of the primary assessment of quantitative content validity and reliability of the IMOS-specific questionnaires. The questions with unacceptable validity and reliability indexes were corrected

**Questionnaire**	**Quantitative content validity**		**Reliability**	
	**Range of questions’ CVI**^a^	**Range of questions’ CVR**^b^	**Internal consistency** **(Cronbach's alpha)**	**Intra-rater reliability**^c^
Osteoporosis diagnosis	1	0.67—1	0.66	0.81 – 0.93
Osteoporosis treatment adherence	0.61—1	0.67—1	0.56	0.68—1
Fall risk assessment	0.94—1	0.67—1	0.54	0.91—1
Fracture and osteoporosis history	0.78—1	0.67—1	0.61	0.72—1
Modified Oswestry	0.94—1	0.67	0.93	0.75 – 1
Muscle health	1	0.67	0.77	1
Reproductive history	0.89—1	0.67—1	-	0.84—1
Exposure to sunlight	0.89—1	0.67—1	0.63	0.83—1
Knowledge about osteoporosis	1	1	0.86	0.61—1
Medical history	0.61—1	0.67—1	-	0.64—1
Health utilization	1	0.67—1	0.64	1

^a^ Content Validity Index

^b^ Content Validity Ratio

^c^ Range of questions’ Kappa coefficient of agreement or Intra Class Correlation (ICC)

##### Physical examinations

Specific physical examinations of the IMOS study were performed by the IMOS questioner after conducting interview at the BMD centers. These examinations included (i) walking speed, (ii) anthropometric measurements (including neck circumference in the middle of the neck height in front (below the laryngeal ridge or apple in men) and in the middle of the cervical vertebrae in the back; right wrist circumference at the top of the lister tubercle of the radius and ulna distal; and right leg circumference in the largest environment), (iii) muscle strength, and (iv) 5 times sit to stand test.

To measure walking speed, the participant normal walking time on a 4.57-m path was recorded by a digital chronometer. The path had been marked with a colored bar. The muscular strength of each hand was measured with a calibrated dynamometer (twice for each hand).To do this, the person should sit upright and in harmony with the dynamometer, so that the device stayed easily on the hand, the shoulder was pulled inwards and the elbow was at a 90-degree angle. The forearm and wrist was in a neutral and comfortable position. In the 5 times sit to stand test, the hands was on the chest and the person got up from the chair 5 times without the help of the hands and the test time was recorded using the chronometer.

##### Bone mineral density

BMD was measured using DXA in the lumbar vertebrae (L1 to L4), total hip, and femur neck according to the standard protocol using HOLOGIC (Discovery and Horizon) devices. Body composition assessments were also done in the same session with BMD by whole-body DXA, vertebral fractures assessment scan, and trabecular bone score. Trained operators were responsible for all scans.

### Training and quality assurance

All people who were involved in the measurements were accordingly trained in both STEPs and IMOS surveys. In the STEPs study, Training Of Trainers (TOT) workshop was held and the interviewers were trained by the trainers. In the IMOS study, all operators of BMD devices were directly trained by a single expert who was cooperating from the formal exclusive representative of HOLOGIC devices in Iran. In the IMOS workshops, the process of conducting the specific section of the IMOS study, method of setting up the data collection centers, method of inviting participants and completing questionnaires, performing physical examinations, completing study forms, measuring BMD, and monitoring and supervising of the study were taught.

### Monitoring and supervision

In this study, monitoring and supervision consisted of two parts: (1) monitoring of sampling method, questioning, clinical examinations, laboratory tests, data collection and entry, and information technology related to the parts integrated with the STEPs study, and (2) monitoring of specific questioning and clinical examinations of the IMOS study and BMD. The first part was monitored through online monitoring of the supervising panel in the study software by the national and university supervisors and face-to-face inspection of data collection processes and completion of the monitoring checklists by the supervisors of the districts.

The specific section of the IMOS study was monitored at two levels by the national and university supervisors. National supervisors monitored the sending and receiving of study equipment, the data collection process, and all stages of the study through the online supervising panel and monitoring checklists. University supervisors also monitored the completeness and flawless of the received equipment, setting-up the data collection centers, obtaining written consent, questioning, physical examinations, and measuring BMD, etc., through face-to-face visits and completing monitoring checklists.

### Data management

Data collection of the STEPs and IMOS studies were performed electronically using web-applications specifically designed for each of studies. At the end of the IMOS data collection process in each of the BMD centers, the operator received the results of each person's scans (as PDF files) along with the data file of all participants (as MS Access file) from the BMD center computer and via flash memory or tablet Secure Digital (SD) card and delivered them to the coordinator of the medical universities.

After completing the data collection process, the data will be downloaded from the studies software and then cleaned. Data cleaning is done in three steps: (i) screening, (ii) detecting possible errors, and (iii) correcting suspicious data. In the screening step, checking the questionnaires using a specific algorithm, validation assessment of the data entry, checking the distribution of variables using the box, histogram, or scoter plots, checking the frequency distribution, various variables contingency tables, and descriptive statistics, and the determination statistically extreme and outlier data and inliers are done. In the second step, suspicious data is divided into four groups: error, true extreme and outlier values, normal and correct values, idiopathic (no reason is given for the error, but the data is still suspicious). Finally, in the third step, we will have three solutions for the suspicious data: correction, deletion, or unchanged. Missing data on gender and age, duplicate data and biologically irrational data will be removed.

### Statistical analysis

We will estimate the prevalence of osteoporosis, osteopenia, and sarcopenia with their 95% Confidence Intervals (CI) at the national level by urban/rural area of residence, gender, and age groups. To obtain the prevalence estimates, we will use the following three weights:

(i) The weight of the correcting differences in the age, gender, and urban/rural distribution of the study sample with their distribution in the population of the province (W_1_ or post-stratification weight). This weight is the inverse of the ratio of the number of samples determined for each age-gender-urban/rural category by the population of that category in each selected province based on the population projection for 2021 by the Statistics Center of Iran;
(ii) The weight of the responding (W_2_). This weight will use to correct the effect of non-responses on the prevalence estimates [24]. Since the variables age, gender, urban/rural area, and stratum of residence are associated with participation in the study, W_2_ will obtain by dividing the number of determined samples by the number of participants (inverse of the probability of responses) in each of the age-gender-urban/rural categories for each province.
(iii) The sampling weight (W_3_). It will be calculated by dividing the number of the population of each stratum by the sample size of that stratum (selected province of the stratum) [25].

To calculate the national prevalence estimates, W_3_ will be multiplied by W_1_ and W_2_.

## Discussion

The strengths of the present IMOS survey in comparison with previous studies are: (i) based on the sampling technique, the study sample is representative of the population over 50 years of age in Iran and it was also sampled from the rural and small towns unlike the previous rounds of IMOS which was only from big cities. (ii) Using the same DXA devices to measure BMD. (iii) TBS assessment, (iv) estimating prevalence of vertebrae fractures using Vertebral Fractures Assessment (VFA), and (v) muscle health assessment and estimating sarcopenia prevalence.

The most important limitation of the study is the possibility of a high non-response rate due to the need for a long travel to reach data collection centers in most cases and synchrony with the COVID-19 pandemic. Encouragement to participate in the study, payment of travel allowance, flexibility in providing BMD centers visit time, and conducting the study after massive vaccination against COVID-19 for the ≥ 50 year age group, are some of the strategies that are considered to overcome this limitation. Weighting data will be done based on the inverse of responding probability in the age and sex subgroups during statistical analyses in order to reduce the effect of non-response on the results.

The IMOS would provide unique and valuable information about the prevalence of osteoporosis and sarcopenia and its determinants at the national level, which can be used for policymaking in the field of musculoskeletal diseases and as the baseline for evaluating the effects of the policies and interventions which might be implemented in the future.

## Data Availability

The datasets of the study will be available from the corresponding author on reasonable request, but restrictions will be placed on the availability of data, which will be used under license for the current study, and so will not be publicly available.

## Abbreviations

IMOS: Iranian Multi-center Osteoporosis Study
NCD: None Communicable Diseases
DXA: Dual-energy X-ray Absorptiometry
TBS: Trabecular Bone Score
BMD: Bone Mineral Density
WHO: Word Health Organization
GPAQ: Global Physical Activity Questionnaire
HDL: High-Density Lipoprotein
ALT: Alanine transaminase
HbA1C: Hemoglobin A1C
FPG: Fasting Plasma Glucose
TSH: Thyroid-Stimulating Hormone
PTH: Parathyroid Hormone
SF-12 questionnaire: The12-Item Short Form Health Survey questionnaire
CVI: Content Validity Index
CVR: Content Validity Ratio
ICC: Intra Class Correlation
TOT: Training of Trainers
SD card: Secure Digital card
CI: Confidence Intervals
VFA: Vertebral Fractures Assessment
NIMAD: Iranian National Institute for Medical Research Development
MOHME: Ministry of Health and Medical Education
